# Right-sided infective endocarditis in association with a left-to-right shunt complicated by haemoptysis and acute renal failure: a case report

**DOI:** 10.1186/s12872-020-01772-y

**Published:** 2020-11-23

**Authors:** Rubi Stephani Hellwege, Meinrad Gawaz

**Affiliations:** Department of Cardiology and Angiology, University Hospital, University of Tübingen, Tübingen, Germany

**Keywords:** Case report, Right-sided endocarditis, Ventricular septal defect, Gerbode defect, Tricuspid valve, *Staphylococcus aureus*, Glomerulonephritis, Haemoptysis, Septic pulmonary embolisms

## Abstract

**Background:**

Infective endocarditis has a relevant clinical impact due to its high morbidity and mortality rates. Right-sided endocarditis has lower complication rates than left-sided endocarditis. Common complications are multiple septic pulmonary embolisms, haemoptysis, and acute renal failure. Risk factors associated with right-sided infective endocarditis are commonly related to intravenous drug abuse, central venous catheters, or infections due to implantable cardiac devices. However, patients with congenital ventricular septal defects might be at high risk of endocarditis and haemodynamic complications.

**Case presentation:**

In the following, we present the case of a 23-year-old man without a previous intravenous drug history with tricuspid valve *Staphylococcus aureus* endocarditis complicated by acute renal failure and haemoptysis caused by multiple pulmonary emboli. In most cases, right-sided endocarditis is associated with several common risk factors, such as intravenous drug abuse, a central venous catheter, or infections due to implantable cardiac devices. In this case, we found a small perimembranous ventricular septal defect corresponding to a type 2 Gerbode defect. This finding raised the suspicion of a congenital ventricular septal defect complicated by a postendocarditis aneurysmal transformation.

**Conclusions:**

Management of the complications of right-sided infective endocarditis requires a multidisciplinary approach. Echocardiographic approaches should include screening for ventricular septal defects in patients without common risk factors for tricuspid valve endocarditis. Patients with undiagnosed congenital ventricular septal defects are at high risk of infective endocarditis. Therefore, endocarditis prophylaxis after dental procedures and/or soft-tissue infections is highly recommended. An acquired ventricular septal defect is a very rare complication of infective endocarditis. Surgical management of small ventricular septal defects without haemodynamic significance is still controversial.

## Background

The diagnosis and management of patients with infective endocarditis (IE) require extensive clinical assessment, advanced cardiac imaging, and an interdisciplinary approach to decrease morbidity and mortality. Right-sided IE has a lower prevalence (10–15%) with lower complication rates than left-sided endocarditis [1, 2]. In most cases, *Staphylococcus aureus* is identified as the pathogen in blood cultures, and the management is conservative with specific antibiotic therapy [1, 2]. Common complications of right-sided IE are haemoptysis caused by septic pulmonary embolisms and acute right-heart failure due to tricuspid regurgitation [1, 2]. Another systemic complication related to *S. aureus* infection is acute diffuse glomerulonephritis caused by immune complex formation and complement C3 deposits in the glomeruli [3–6]. Right-sided endocarditis is commonly associated with intravenous drug abuse, central venous catheters, and implantable cardiac device infections. However, patients with a congenital ventricular septal defect (VSD) are at high risk of IE [7–10]. Echocardiographic findings, such as a left-to-right shunt in IE, should always raise the suspicion of an acquired VSD, principally in previous young and healthy patients [10–14]. In the literature, several cases of left ventricular-to-right atrial shunt, also known as the Gerbode defect [15], were reported in association with IE [12–14, 16–18].

## Case presentation

A 23-year-old man was referred from another hospital with a history of a dry cough, fever (> 39 °C), and malaise, mostly at noon and at night. The symptoms started after he developed a self-limited skin and soft tissue infection on his left hand two weeks ago. Initially, he was hospitalized for 3 days under suspicion of COVID-19 infection. Empiric antibiotic therapy with piperacillin-tazobactam was started after his admission. Transthoracic echocardiography revealed floating vegetation (35–40 mm) on the tricuspid valve. Prior to transfer to our clinic, he also complained of blood-stained sputum and two episodes of diarrhoea and vomiting. The patient originally came from Romania, is a construction worker, and denied intravenous drug abuse. However, he admitted to having contact with a patient with active pulmonary tuberculosis in the past.

On physical examination at admission, his vital signs showed a blood pressure of 128/60 mmHg, a heart rate of 112 bpm, oxygen saturation of 97% on room air and a subfebrile temperature (37.5 °C). Cardiac auscultation revealed a grade III/VI holosystolic murmur over the tricuspid valve. There were also bilateral rales and crackles audible at the base of the lungs and right basal dullness on percussion. Examination of his extremities and skin revealed bilateral ankle pitting oedema and an isolated left-hand oedema with concomitant swelling of the third metacarpal-phalangeal joint. Neurological examination was unremarkable.

Initial investigations included laboratory tests and blood culture sets. An electrocardiogram showed a sinus rhythm without signs of underlying ischaemia or atrioventricular block. An initial full blood count revealed mild leucocytosis, neutrophilia, left deviation, and microcytic anaemia (Table 1). High levels of C-reactive protein and procalcitonin suggested a bacterial infection. In addition, the patient presented with a concomitant acute kidney injury (creatinine 1.5 mg/dl, BUN 112 mg/dl). Liver function tests showed elevated levels of alkaline phosphatase and gamma-GT and low levels of cholinesterase (Table 1). Empirical antibiotic therapy with ampicillin, flucloxacillin and gentamicin, according to the current European Guidelines for the empirical treatment of native valve endocarditis, was administered [1]. A throat swab for the SARS-CoV-2-RNA PCR test was reported to be negative.

**Table 1:** Initial laboratory investigations

Test	Result	Normal range
*Full blood count (FBC)*		
Leucocyte count	16,540 1/µl	3800–10,300
Erythrocyte count	3.05 Mio/µl	4.2–6.2
Haemoglobin	8.7 g/dl	14–18
Haematocrit	24.2%	42–52
MCH	28.5 pg	27–34
MCHC	36.0 g/dl	32–36
MCV	79.3 fl	80–93
Thrombocyte count	211 × 10^9^/L	150–450
C-reactive protein (CPR)	21.24 mg/dl	max. 0.50
Procalcitonin	9.75 ng/ml	max. 0.1
ESR 1st hour	57 mm	0–15
Creatinine	1.6 mg/dl	0.6–1.1
GFR–CKD–EPI	60 ml/min/1.73m^2^	> 60
BUN	112 mg/dl	12–46
Albumin	1.3 g/dl	3.0–5.0
*Liver function tests (LFT)*		
AST/GOT	40 U/l	max. 50
ALT/GPT	35 U/l	max. 50
GGT	138 U/l	max. 60
LDH	207 U/l	max. 250
Alkaline phosphatase (ALP)	138 U/l	40–130
Bilirubin total	1.1 mg/dl	max. 1.1
Cholinesterase (CHE)	2.0 kU/l	4.9–12.0

Mean corpuscular haemoglobin (MCH), mean corpuscular haemoglobin concentration (MCHC), mean corpuscular volume (MCV), erythrocyte sedimentation rate (ESR), glomerular filtration rate (GFR), blood urea nitrogen (BUN), aspartate amino transferase (AST), alanine amino transferase (ALT), gamma glutamyl transferase (GGT), lactate dehydrogenase (LDH)

**Fig. 1:**
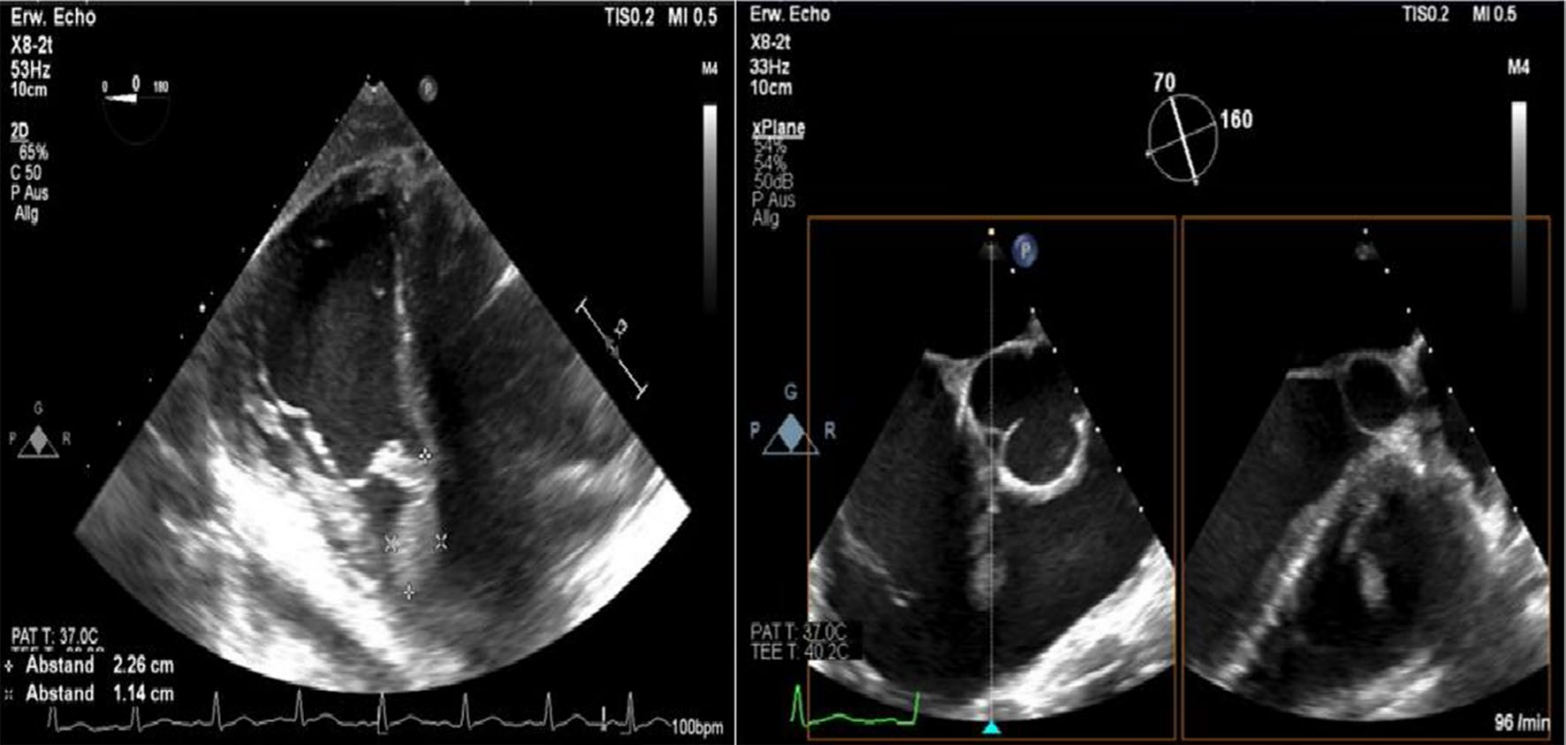
Initial transoesophageal echocardiography showing vegetation on the septal leaflet of the tricuspid valve

**Fig. 2:**
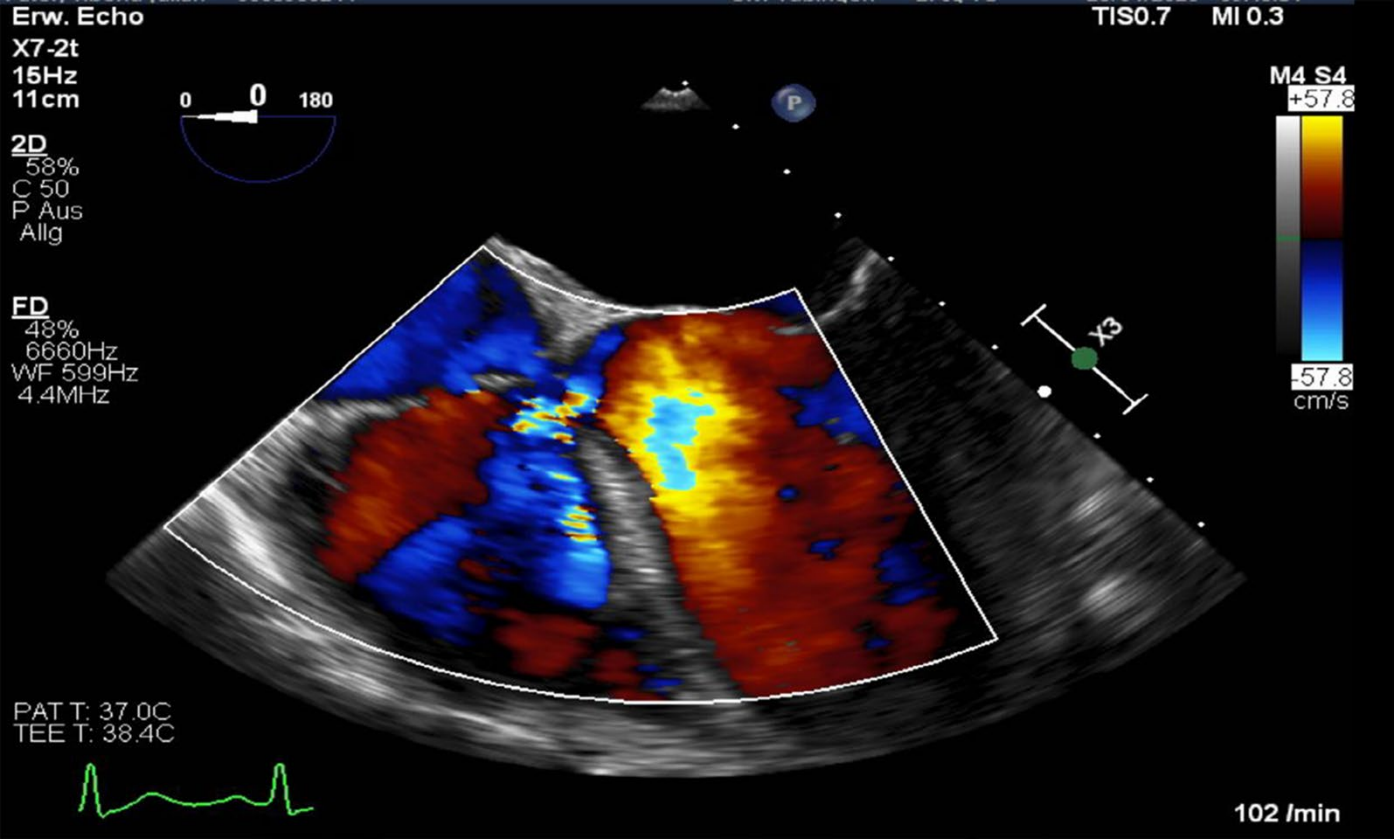
Transoesophageal echocardiography showing a left-to-right shunt in colour Doppler corresponding to a VSD

After admission, we performed transoesophageal echocardiography (Fig. 1, Additional file 1: Video 1), which demonstrated 25 × 15 mm vegetation on the septal leaflet of the tricuspid valve without evidence of severe tricuspid regurgitation. The other valves presented no vegetation or severe regurgitation. The left ventricular ejection fraction was normal, and intracardiac thrombi were not observed. Further relevant echocardiographic findings revealed a normal right ventricular function and pulmonary arterial systolic pressure of 30 mmHg. A small ventricular left-to-right shunt due to a ventricular septal defect was observed on colour Doppler (Fig. 2, Additional file 1: Video 2).

**Fig. 3:**
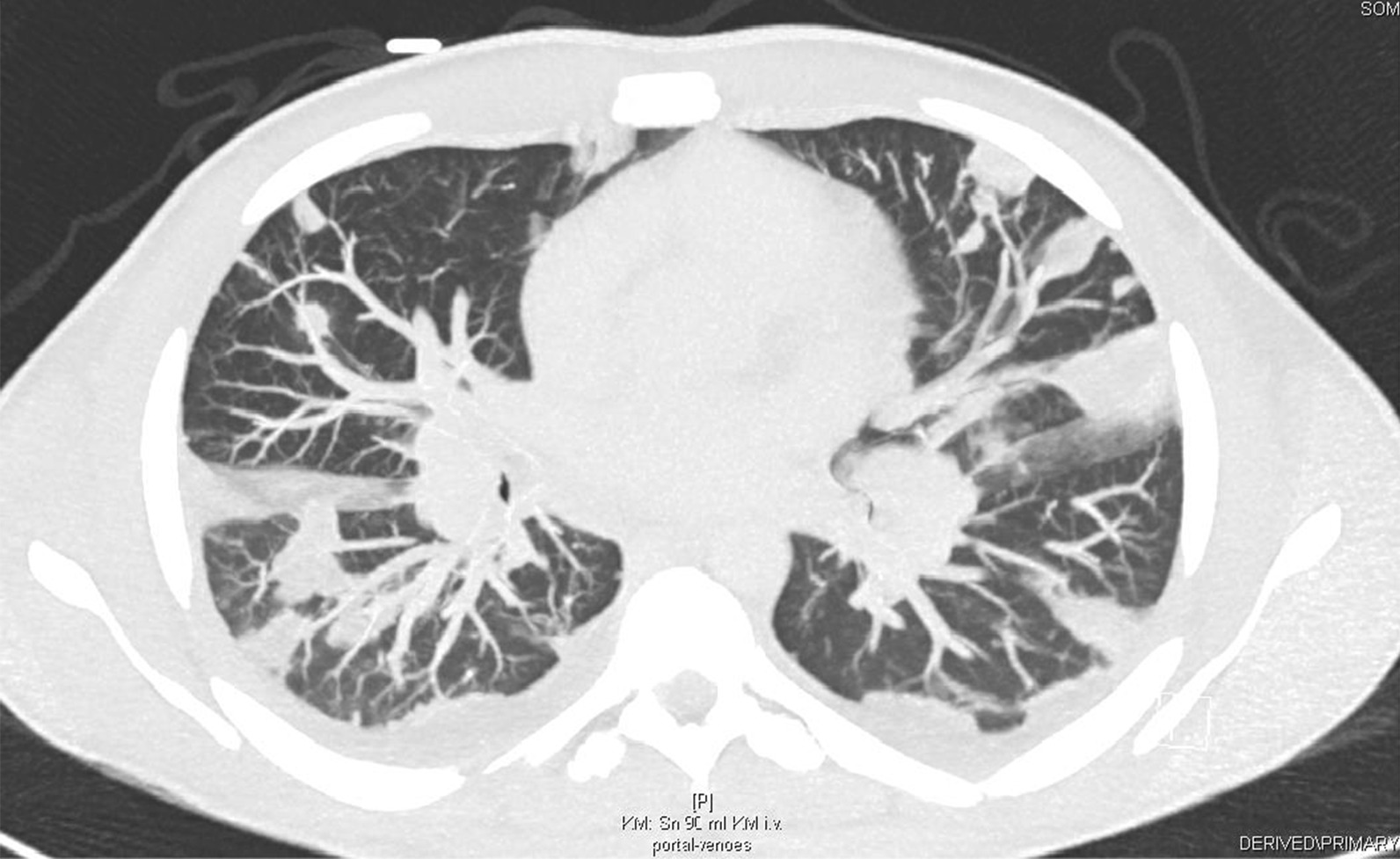
A thorax CT scan demonstrating bilateral pneumonic infiltrates, septic emboli, and pleural effusions

In addition, a CT scan of the thorax and abdomen demonstrated bilateral disseminated multiple septic pulmonary emboli and concomitant pneumonic infiltrates as well as mild bilateral pleural effusions (Fig. 3). On the CT abdomen scan, no additional organ emboli or abscess formations were found. A coronary CT angiography showed no evidence of calcium plaques suggestive of coronary artery disease or malformations. Although this patient did not present neurological symptoms, a brain CT was obtained, which showed normal findings. A CT scan of the left hand revealed a soft-tissue infection or phlegmon without bone involvement.

After admission, at least two blood culture sets were reported to be positive for methicillin-sensitive *Staphylococcus aureus* (MSSA). The final diagnosis of *S. aureus* bacteraemia and isolated right-sided endocarditis was based on two major criteria according to the modified Duke criteria for endocarditis [1]. We continued administering flucloxacillin, following the current guidelines [1]. Clarithromycin was started because of atypical pneumonic infiltrates on CT, and a course of 10 days of ceftazidime was also added to the antibiotic regimen due to concern about a *Pseudomonas sp.* infection. Previously, the initial antibiotic therapy with gentamicin was stopped in consideration of his acute kidney injury. A therapeutic intravenous anticoagulation with unfractionated heparin was begun after the diagnosis of septic pulmonary emboli.

**Fig. 4:**
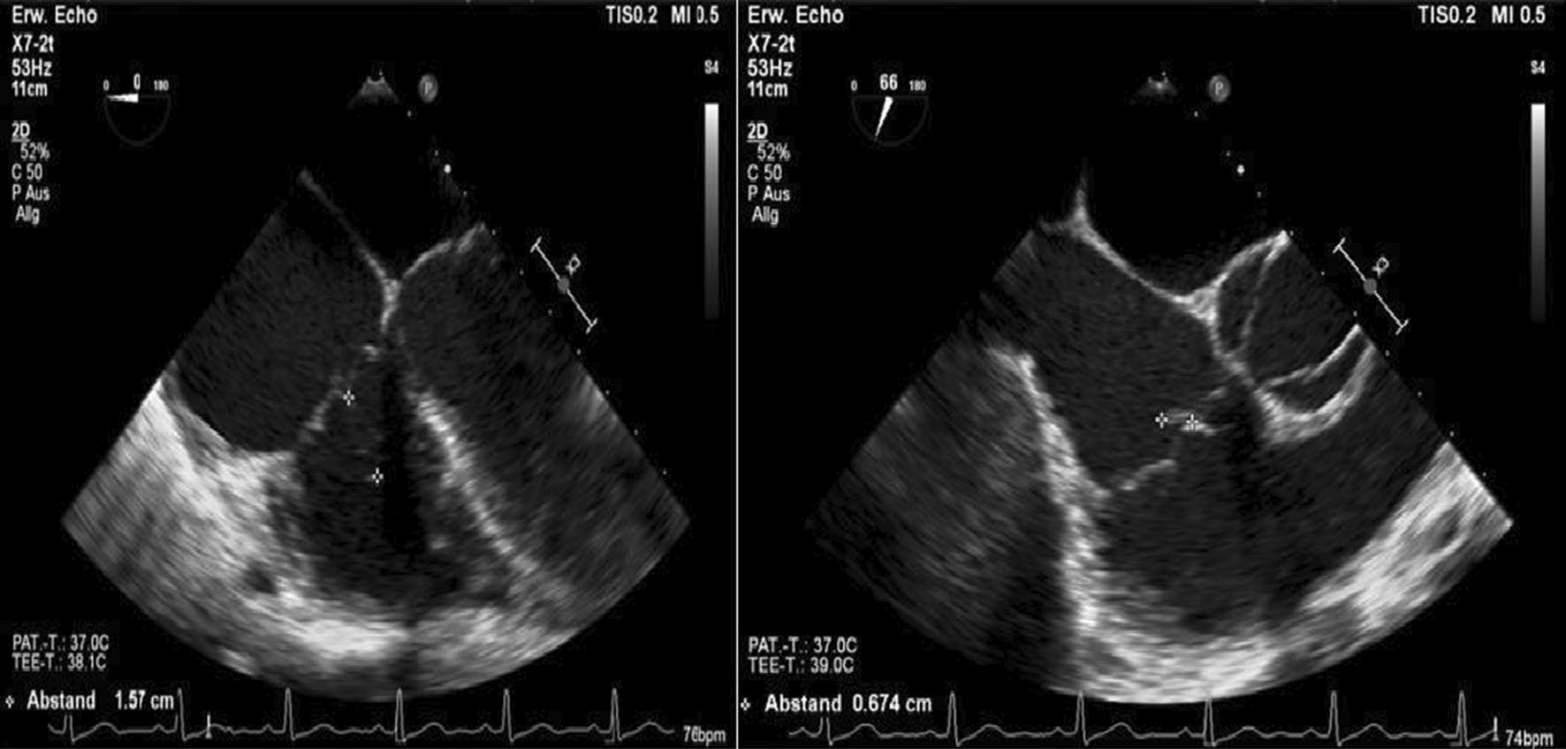
Transoesophageal echocardiography showing a decrease in vegetation on the septal leaflet of the tricuspid valve

Furthermore, a second transoesophageal echocardiography was performed 10 days after starting antibiotic therapy (Fig. 4). Fortunately, it showed diminished vegetation (16 × 7 mm) on the septal leaflet of the tricuspid valve, without any evidence of further valve lesions. The left-to-right shunt due to the ventricular septal defect was stable without haemodynamic significance. In consensus with our endocarditis team, including a cardiac surgery evaluation, we decided on conservative management of the isolated right-sided endocarditis.

During hospitalization, the patient gained weight and there was an increase in peripheral oedema and anasarca, and increased levels of creatinine, BUN, and hypoalbuminemia were noted on laboratory tests (Table 1). In addition, urine diagnostic tests revealed macrohematuria, albuminuria, high levels of A1-microglobulin, and a high protein-creatinine ratio, suggesting acute tubular injury (Table 2). Autoantibodies and C3 complement tests were conducted, which revealed a low C3 level and a negative ANCA titre (Table 3). In consideration of these findings, we suspected glomerulonephritis associated with *S. aureus* infection and decided to not perform a renal biopsy. Instead, we administered diuretic therapy with amiloride and hydrochlorothiazide to treat the anasarca symptoms. In addition, relevant proteinuria was observed on his 24-h urine protein test (Table 2). Consequently, we started a regimen of corticosteroids with prednisolone therapy (1 mg/kg BW) for 4 weeks, and then it was tapered weekly, showing a gradual improvement in the patient’s proteinuria and oedema (Table 2).

**Table 2:** Urine diagnostic tests

Test spot urine sample	On admission	At follow-up (4 weeks later)	Normal range, units
Protein	7.18	1.19	< 0.10 g/L
Creatinine	86	158	mg/dL
Protein/creatinine ratio	8349	753	< 100 mg/g
Albumin	4420	742	< 20 mg/L
A1-microglobulin	529	21	< 13 mg/L
A1-microglobulin/creatinine ratio	615.1	13.3	< 13.0 mg/g
A2-macroglobulin	12.5	< 2.3	< 2.4 mg/L
IgG	1850	127	< 10 mg/L
IgG/creatinine ratio	2151.2	80.4	< 10 mg/g
*24-h urine test*			
Creatinine /24 h	1221		800–2000 mg/24 h
BUN /24 h	16,724		5500–22,000 mg/24 h
Protein /24 h	9.95		max. 0.15 g/24 h
Protein/creatinine ratio	8152		max. 100 mg/g
A1-microglobulin	105		max. 13 mg/L
A2-macroglobulin	15.1		0–2.4 mg/L
Albumin /24 h	5698		max. 30 mg/24 h

Immunoglobulin G (IgG), blood urea nitrogen (BUN)

**Table 3:** Autoantibodies tests

Test	Result (normal range)
Anti-GMB antibodies	3.1 U/ml (< 7)
ANA	1: < 80 U/ml, negative
*ANCA*	
cANCA, pANCA	1: < 10 U/ml, negative
C3—Complement	80 mg/dl (90–180)
C4—Complement	14 mg/dl (10–40)

Antinuclear antibody (ANA), anti-neutrophil cytoplasmic antibody (ANCA), anti-glomerular basement membrane antibodies (anti-GMB)

**Fig. 5:**
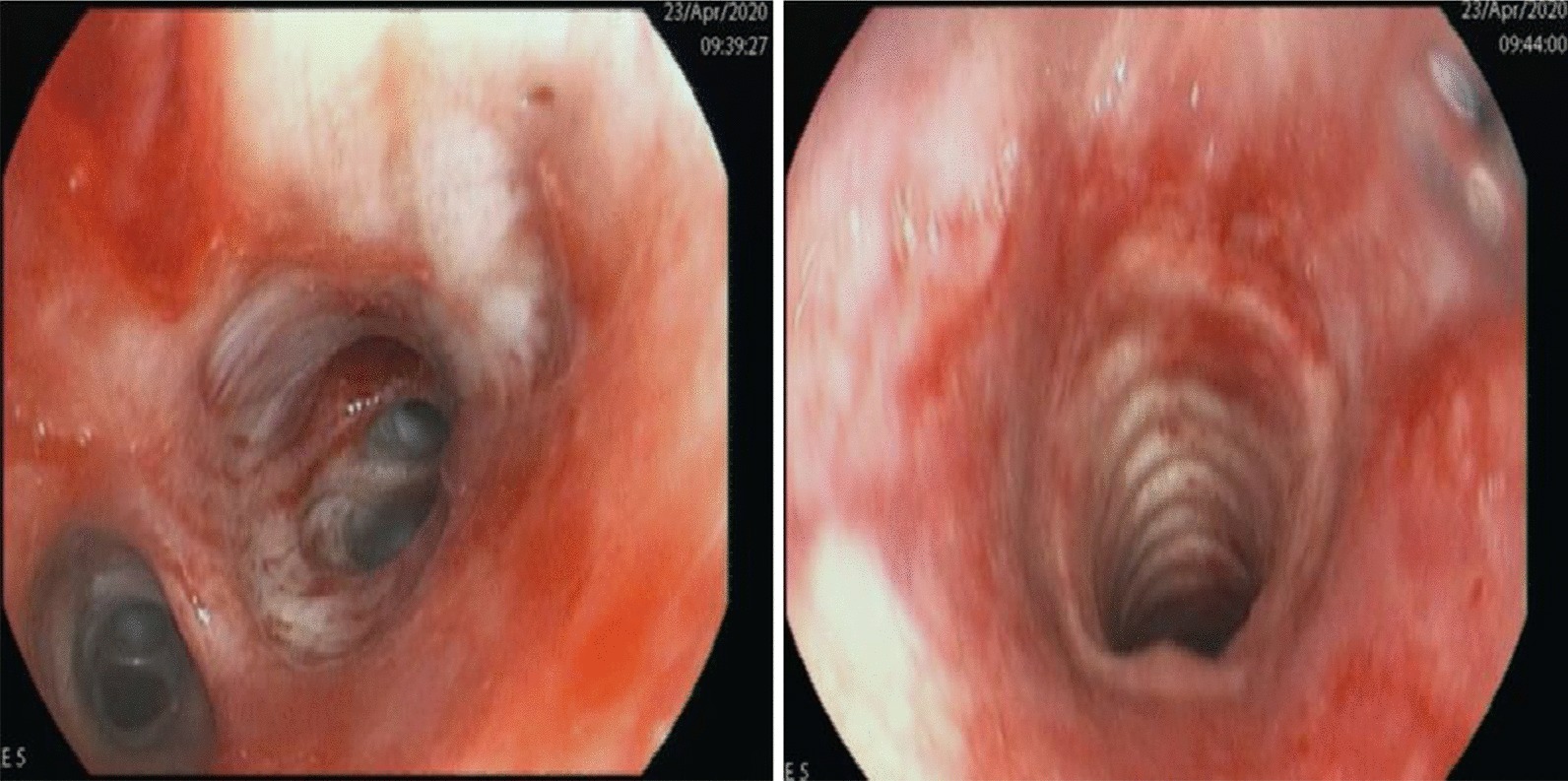
Bronchoscopy showing tracheobronchitis and diffuse bleeding of the lower right lobe

After an acute episode of massive haemoptysis, we stopped the intravenous anticoagulation and performed an urgent bronchoscopy (Fig. 5). It showed tracheobronchitis with diffuse bleeding in segment 8 of the lower right lobe requiring an endobronchial tamponade for 24 h to stop the bleeding. Subsequently, the patient underwent a revision bronchoscopy for extraction of the endobronchial tamponade. It showed abundant purulent bronchial secretions without signs of de novo active bleeding. Acid-fast stain tests, *Pneumocystis jirovecii*, and respiratory viruses (RSV-RNA, Influenza A, B) were negative in the bronchoalveolar lavage. Although the gamma-interferon test was positive for a latent tuberculosis infection, we considered it an isolated finding not relevant to diagnose active pulmonary tuberculosis. Haemoptysis episodes are more likely due to multiple septic pulmonary emboli and concomitant *S. aureus* pneumonia, as previously found on the patient’s CT-thorax scans.

Further investigations related to explaining the microcytic anaemia revealed an iron deficiency (iron 33 µg/dL, ferritin 116 mcg/dL, transferrin 78 mg/dL). However, this finding might be explained as a combination of nutrition deficiency, recurrent haemoptysis, and inflammatory systemic disease due to acute infective endocarditis. His other parameters were at normal levels without suspicion of haemolysis or vitamin B12 deficiency.

**Fig. 6:**
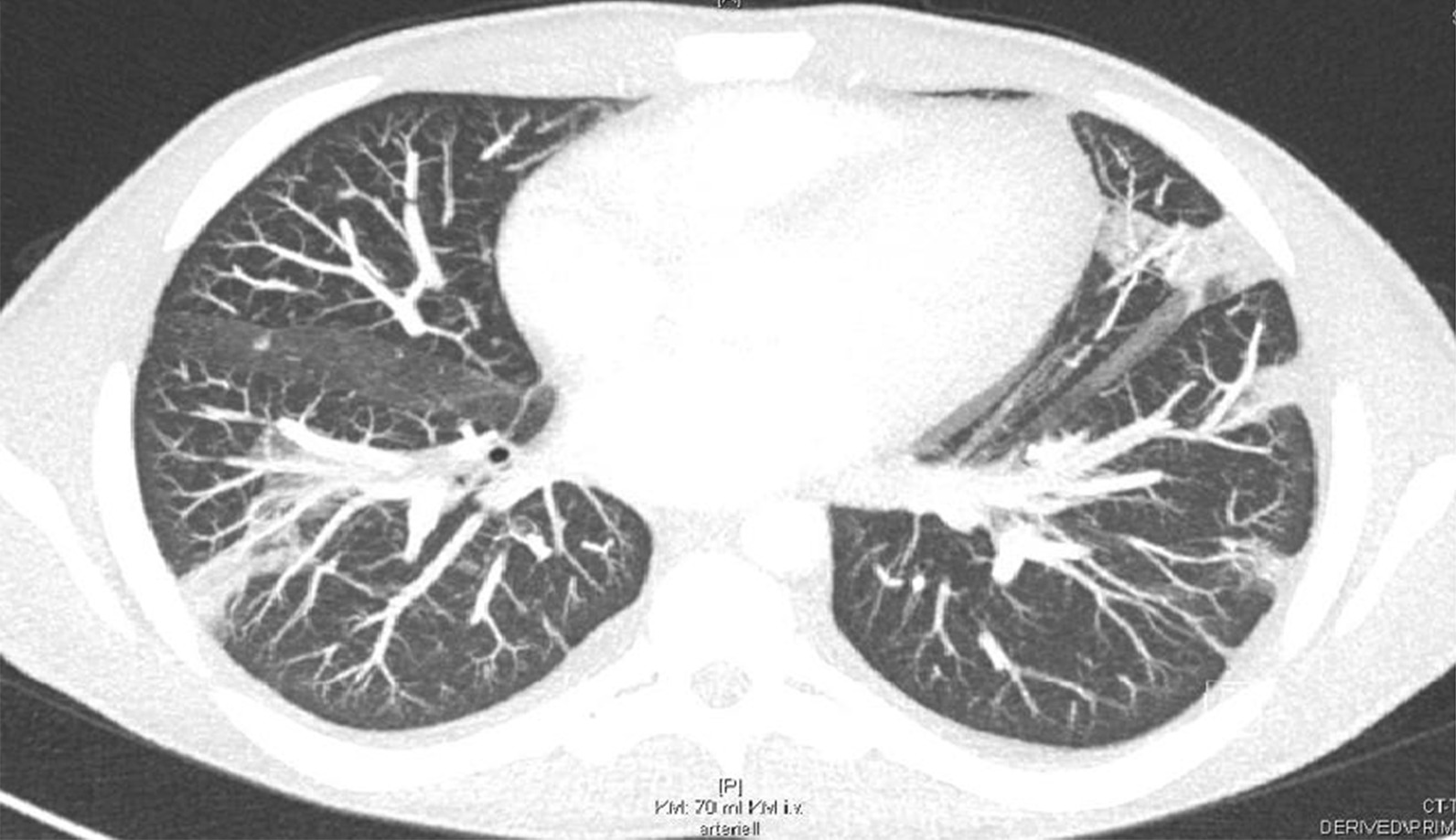
A follow-up thorax CT scan demonstrated fewer pneumonic infiltrates and septic pulmonary emboli

Prior to discharge, a thorax CT scan revealed remission of the pneumonic infiltrates and septic emboli after two weeks of antibiotic therapy (Fig. 6). After 4 weeks of hospitalization, considerable improvement of renal function, proteinuria, and inflammatory markers was observed. The patient was discharged with combined antibiotic therapy of flucloxacillin and clarithromycin at 4 weeks.

**Fig. 7:**
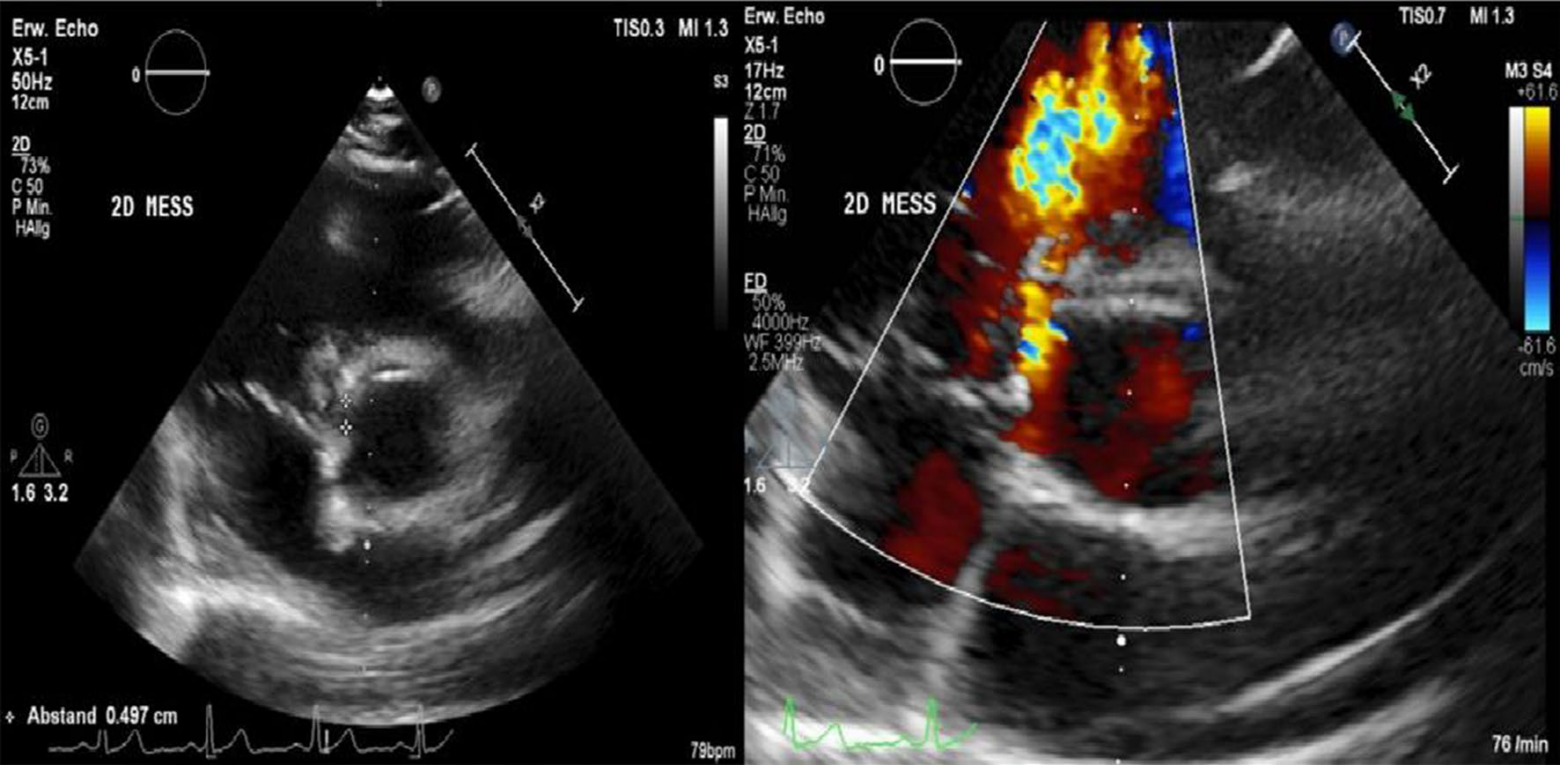
Transoesophageal echocardiography at the 4-week follow-up showed a 5 mm VSD and a left-to-right shunt

**Fig. 8:**
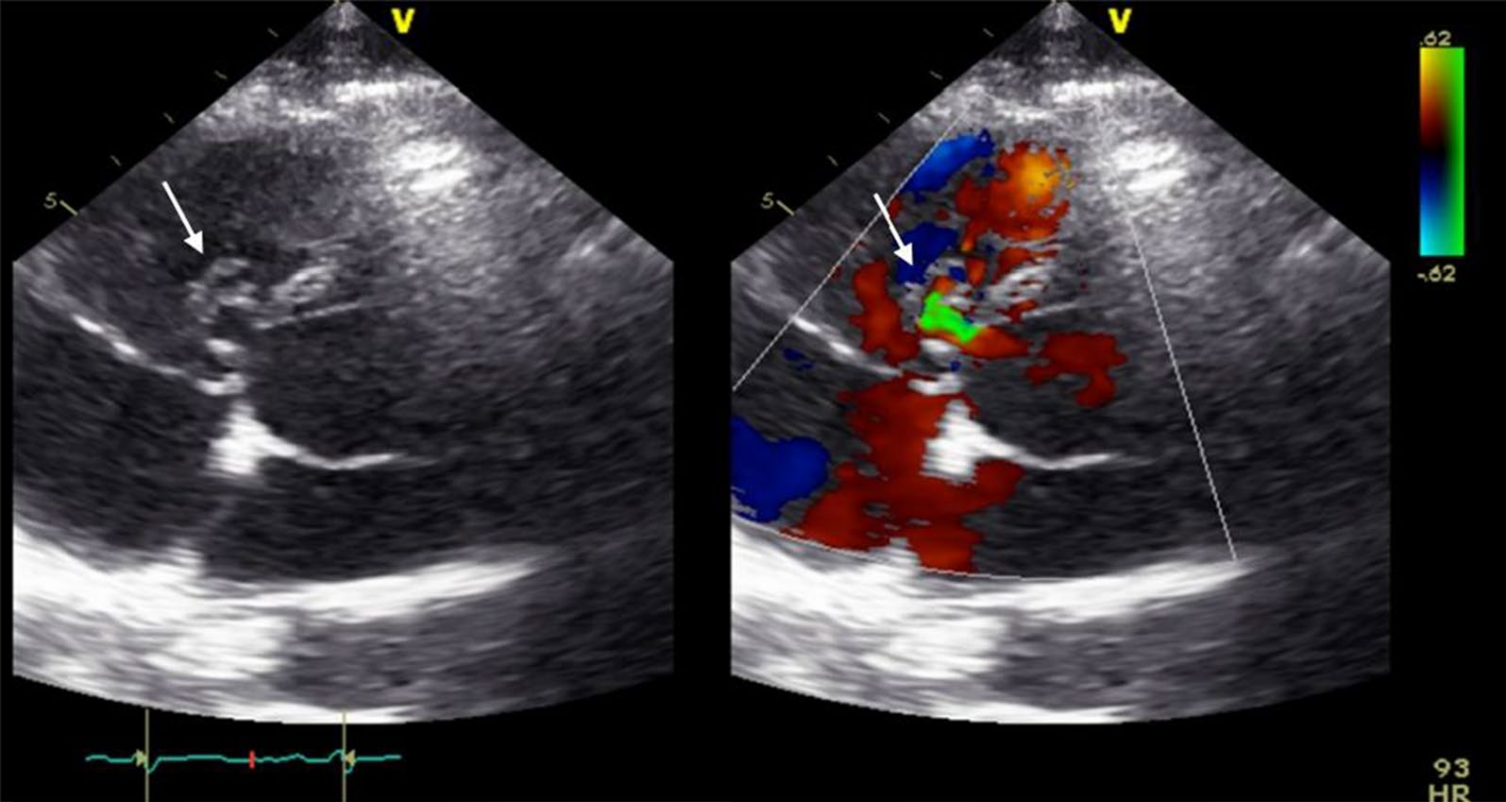
Aneurysmal transformation of the VSD with involvement of the septal leaflet of the tricuspid valve

**Fig. 9:**
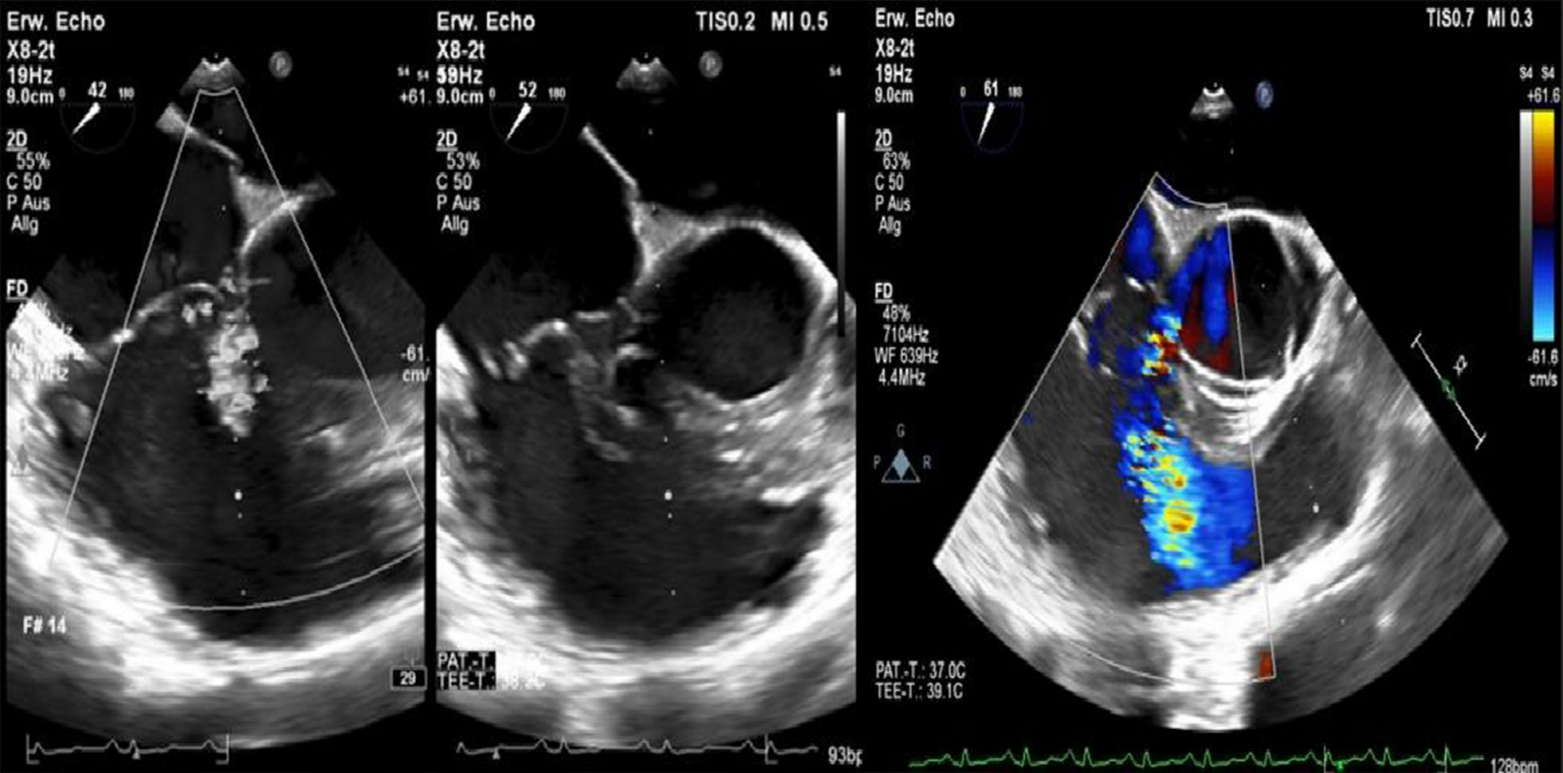
Transoesophageal echocardiography showing a VSD corresponding to a type 2 Gerbode defect

At follow-up, one month after discharge, the patient presented with a good recovery of renal function and proteinuria (Table 2). Laboratory tests were unremarkable. Additionally, transoesophageal echocardiography showed no more relevant vegetation of the tricuspid valve only some mild regurgitation (Fig. 7). However, an aneurysmal transformation of the ventricular septal defect (5 mm) located infravalvular to the septal leaflet of the tricuspid valve was found (Fig. 8, Additional file 1: Video 3). The left-to-right shunt was not haemodynamically significant (systolic velocity of 5.5 m/s) without involvement of the aortic valve or aorta (Fig. 9). After surgical evaluation, conservative management, including endocarditis prophylaxis, was continued. The patient was scheduled for further echocardiographic follow-up and cardiac catheterization to determine the invasive haemodynamic parameters.

## Discussion and conclusions

Ventricular septal defects (VSDs) with left-to-right ventricular shunts are frequently congenital and are associated with a higher incidence of endocarditis in comparison with patients without congenital ventricular septal defects [7–10]. Acquired VSD after an episode of endocarditis has been previously described in several case reports as a very rare complication [12–14, 16–18]. The Gerbode defect is a perimembranous VSD with a secondary left ventricular-to-right atrium shunt [10, 15]. Gerbode was an American cardiac surgeon who successfully reported the first surgical management of five patients with VSD and left-to-right atrial shunt in 1958 [15].

**Fig. 10:**
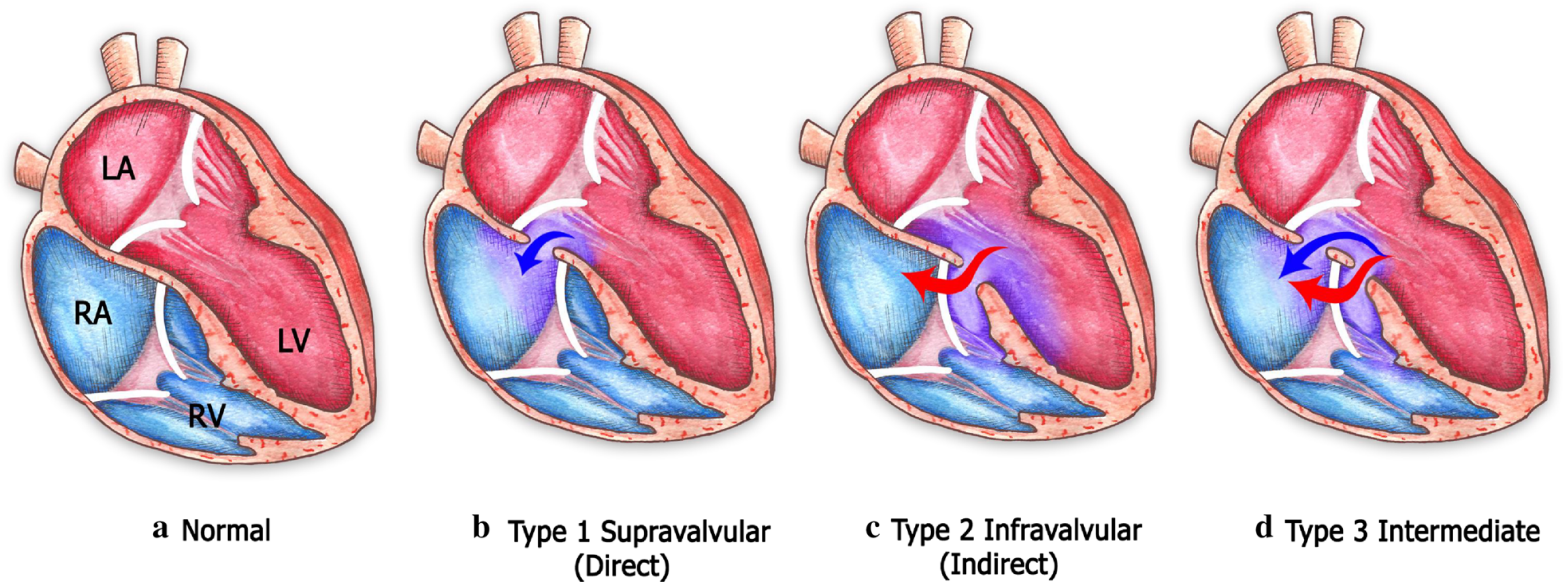
Classification and anatomical features of the 3 types of the Gerbode defect. (Illustration by Ivonne Hernández del Muro © 2020)

Classical features of the Gerbode defect are communication between the left ventricle and the right atrium through a ventricular septal defect localized supra- or infravalvular in anatomical relation to the septal leaflet of the tricuspid valve [10, 15, 19]. According to the classification described by Perry et al. [19], a type 1 Gerbode defect consists of a left-ventricular-to-right-atrium shunt localized supravalvular to the tricuspid valve (Fig. 10). In contrast, in type 2 Gerbode defects, there is a left-ventricular-to-right-ventricular shunt localized infravalvular to the septal leaflet of the tricuspid valve, and owing to tricuspid regurgitation, an indirect left-ventricular-to-right atrium communication develops (Fig. 10). Type 3 Gerbode defects consist of a combination of both supra- and infravalvular left-to right shunts (Fig. 10) [10, 19].

The diagnosis of a ventricular septal defect is based on clinical and echocardiographic findings, such as evidence of cardiac murmurs in association with a left-to-right ventricular shunt on colour Doppler that may be difficult to detect in asymptomatic young patients, who might unknowingly be at a higher risk of endocarditis [7, 9]. Skin and soft tissue infections, as reported in this case, represent a portal of bacterial entry to the blood circulation, causing *S. aureus* endocarditis. However, current European and American guidelines do not routinely recommend endocarditis prophylaxis in patients with acyanotic congenital heart defects, since this population is considered at intermediate risk for infective endocarditis [1, 20].

Infective endocarditis, specifically caused by *S. aureus*, has been related to other common complications, such as nephrotic syndrome and glomerulonephritis [3–6]. The underlying pathologic mechanism is immune-mediated due to the formation of immune complexes and glomerular deposition of complement C3 [3, 5, 6]. In this case, we decided to administer corticosteroid therapy to treat acute diffuse glomerulonephritis secondary to *S. aureus* infection in combination with antibiotic therapy with a satisfactory reduction of proteinuria and gradual improvement of the patient’s renal function.

Finally, ventricular septal defects complicated by endocarditis without haemodynamic significance are commonly treated conservatively with endocarditis prophylaxis to avoid further endocarditis episodes. Successful surgical management of small ventricular septal defects without haemodynamic significance after endocarditis has been reported in several cases [10, 21–24].

Right-sided infective endocarditis in patients with unknown ventricular septal defects has relevant clinical significance, from diagnosis to management, intervention, and the prevention of further endocarditis episodes. An acquired ventricular septal defect after tricuspid endocarditis is very rare, but it has been described as a possible complication [12–14, 16–18]. Patients with known congenital ventricular septal defects should also be included as high-risk patients in the guidelines to receive appropriate endocarditis prophylaxis. Moreover, severe complications of right-sided endocarditis, including septic pulmonary embolisms with a frequent incidence of haemoptysis and concomitant pneumonia, might require invasive interventions, intensive care management, and mechanical ventilation. Nephrotic syndrome and glomerulonephritis due to acute *S. aureus* infection is a common complication of infective endocarditis [3, 5, 6]. Concomitant antibiotic and corticosteroid therapy might be required to improve proteinuria and renal function, as we described in this case.

In conclusion, patients with right-sided endocarditis often have common risk factors. However, screening for ventricular septal defects is mandatory in patients with a negative history of intravenous drug abuse, implantable cardiac devices, or central venous catheter infections. Current guidelines do not recommend endocarditis prophylaxis in acyanotic heart defects. However, patients with congenital ventricular septal defects are at high risk for infective endocarditis after common bacterial exposure, such as dental procedures and soft-tissue infections, which require endocarditis prophylaxis.

An acquired ventricular septal defect after endocarditis is a very rare complication, but there are many clinical cases reported in the previous literature. Therefore, echocardiographic follow-up is mandatory in all cases. Complications of infective endocarditis should be managed within an interdisciplinary team to reduce morbidity and improve outcomes. Surgical treatment of postendocarditis ventricular septal defects is still controversial in cases without haemodynamic significance.

## Supplementary information

**Additional file 1**. **Video 1**: Transoesophageal echocardiography showing vegetation on the septal leaflet of the tricuspid valve. **Video 2**: Transoesophageal echocardiography showing 378 a left-to-right shunt corresponding to atype 2 Gerbode defect. **Video 3**: Transoesophageal echocardiography showing a type 2 Gerbode defect after tricuspid valve endocarditis.

## Data Availability

All data generated or analysed during this study are included in this published article.

## Abbreviations

IE: Infective endocarditis
VSD: Ventricular septal defect
COVID-19: Coronavirus disease-19
FBC: Full blood count
MCH: Mean corpuscular haemoglobin
MCHC: Mean corpuscular haemoglobin concentration
MCV: Mean corpuscular volume
CPR: C-reactive protein
ESR: Erythrocyte sedimentation rate
GFR: Glomerular filtration rate
BUN: Blood urea nitrogen
AST: Aspartate amino transferase
ALT: Alanine amino transferase
GGT: Gamma glutamyl transferase
LDH: Lactate dehydrogenase
CT: Computer tomography
MSSA: Methicillin-sensitive Staphylococcus aureus
BW: Body weight;
IgG: Immunoglobulin G
ANA: Antinuclear antibody
ANCA: Anti-neutrophil cytoplasmic antibody
Anti-GMB: Anti-glomerular basement membrane antibodies
RSV: Respiratory-syncytial virus
