# Supplementary figures and images for: Right-sided infective endocarditis in association with a left-to-right shunt complicated by haemoptysis and acute renal failure: a case report
Source: BMC Cardiovasc Disord. 2020 Nov 23;20:494. doi: 10.1186/s12872-020-01772-y (PMC7682127; doi:10.1186/s12872-020-01772-y)

## Slide 1
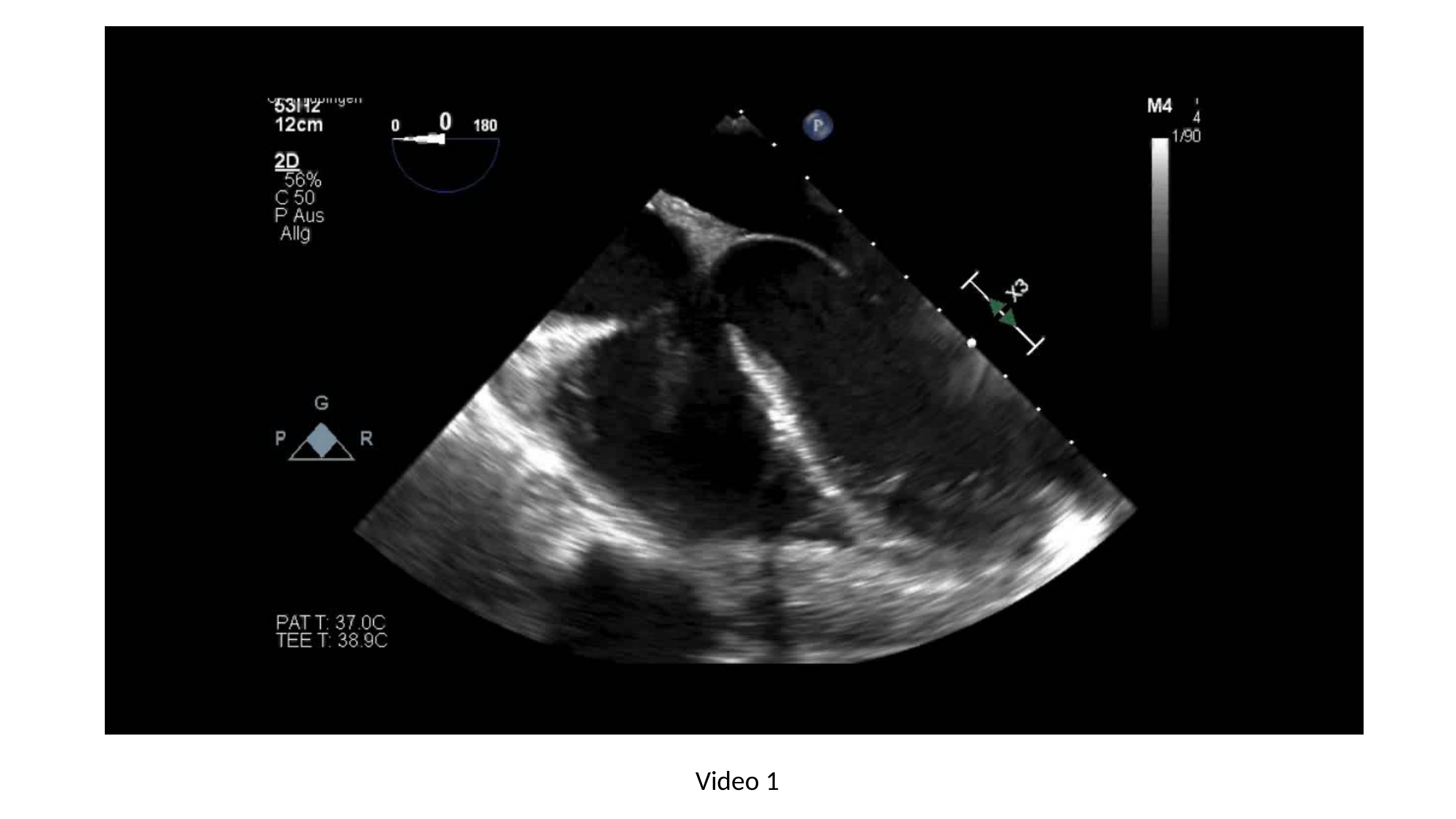

Video 1

## Slide 2
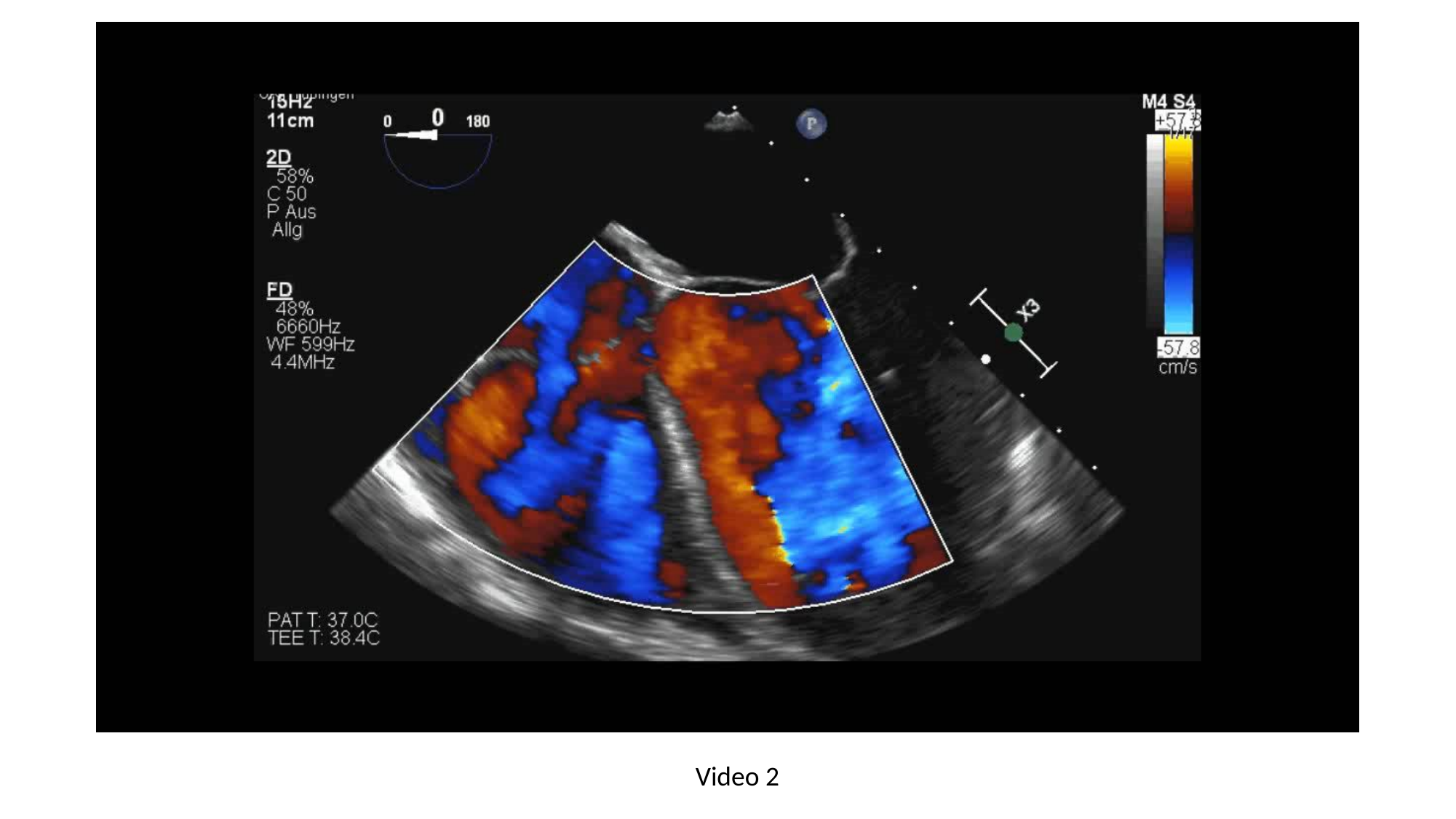

Video 2

## Slide 3
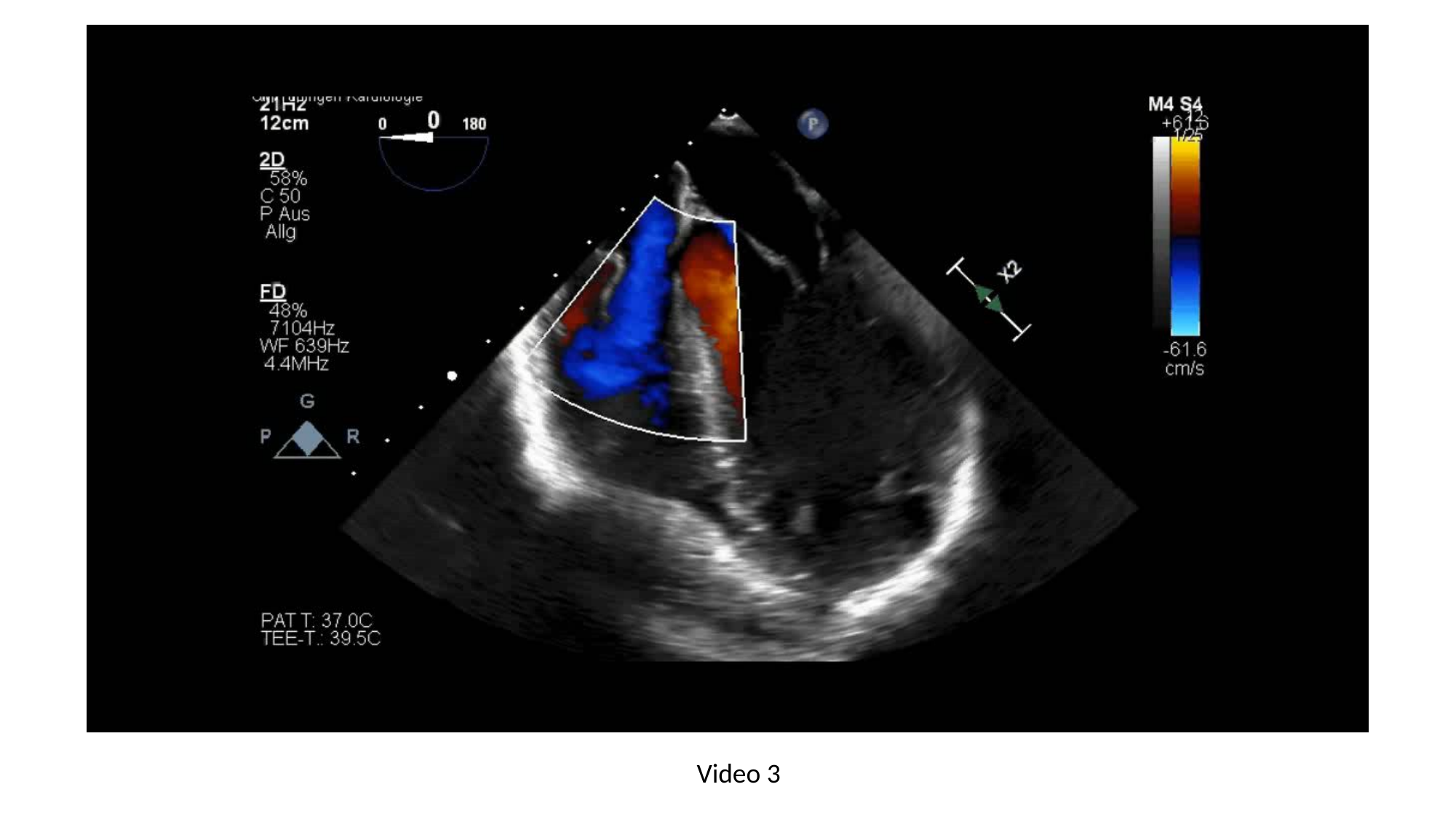

Video 3

Supplement: Supplementary file 1 — Additional file 1. Video 1: Transoesophageal echocardiography showing vegetation on the septal leaflet of the tricuspid valve. Video 2: Transoesophageal echocardiography showing 378 a left-to-right shunt corresponding to atype 2 Gerbode defect. Video 3: Transoesophageal echocardiography showing a type 2 Gerbode defect after tricuspid valve endocarditis. [file 12872_2020_1772_MOESM1_ESM.pptx]
